# Pilot evaluation of the Health Organization and System Trustworthiness scale: reliability and validity testing

**DOI:** 10.1186/s12913-025-12724-7

**Published:** 2025-05-21

**Authors:** Andrew Anderson, Dakota W Cintron

**Affiliations:** 1 Department of Health Policy & Management, Johns Hopkins University, Baltimore, MD USA; 2 Claremont Graduate University, 150 E 10th St, Claremont, CA 91711 USA

**Keywords:** Trust, Trustworthiness, COVID- 19

## Abstract

**Objective:**

This study evaluated the reliability, validity, and measurement invariance of the Healthcare Organization and System Trustworthiness (HOST) scale, a tool designed to assess perceived trustworthiness in healthcare systems.

**Study design:**

Exploratory and confirmatory factor analyses (EFA/CFA) were conducted to examine the scale’s structure, while measurement testing assessed its consistency across demographic groups. Agreement with the Medical Mistrust Index (MMI) was evaluated using a Bland-Altman plot, and concurrent validity was explored through associations with seven outcomes, including vaccine hesitancy, uptake, and trust in government.

**Data collection/extraction methods:**

Data were collected from a national sample of 4,100 U.S. residents aged 18 years and older, with a diverse representation of race/ethnicity (25% Black, 24% Hispanic, 32% White) and LGBTQ identity (13% as cisgendered bisexual, 5% as cisgendered gay/lesbian, and 82% cisgendered straight).

**Findings:**

The HOST scale demonstrated a unidimensional structure, high internal consistency (α = 0.80), and strong concurrent validity with the MMI (*r* = 0.52). Scalar invariance was established across gender, LGBTQ status and regular health care access. Weak invariance was observed across racial groups. Compared to the MMI, the HOST scale had stronger correlations with trust in vaccine-related actors (*r* = 0.71 vs. 0.14) and perceptions of government trustworthiness at the federal (*r* = -0.38 vs. -0.15), state (*r* = -0.40 vs. -0.09), and local levels (*r* = -0.42 vs. -0.11).

**Conclusions:**

The HOST scale shows promise as a tool for assessing trustworthiness in healthcare systems, particularly in capturing perceptions of fairness, competence, and future expectations. While its strong predictive validity across diverse outcomes underscores its potential utility, further refinement and validation are necessary before broader application. Additional testing is recommended to explore its use in addressing health disparities and informing public health strategies.

**Supplementary Information:**

The online version contains supplementary material available at 10.1186/s12913-025-12724-7.

## Introduction

Medical mistrust is a social determinant of health associated with adverse health and healthcare outcomes [1, 2]. It is commonly defined as unease or suspicion towards healthcare providers and medical research institutions [2]. This mistrust often arises from the belief that healthcare professionals may not act in the best interests of patients, especially those from historically marginalized groups [3]. Although various definitions exist, they generally revolve around similar concepts. Medical mistrust significantly affects healthcare utilization, treatment adherence, self-reported health status, patient satisfaction, and participation in clinical trials, with particularly pronounced effects among racial and ethnic minorities, LGBTQ + individuals, and individuals with lower socioeconomic status [4–10]. It was frequently named as a key factor in the low early uptake of vaccines during the COVID- 19 pandemic, particularly among Black Americans [7]. Discrimination, racism, and negative interpersonal experiences in healthcare settings contribute to this mistrust [11, 12]. 

Medical mistrust is rooted in the perceived trustworthiness of healthcare organizations, shaped by direct and indirect experiences with healthcare [13]. One analysis found that approximately 1 in 20 patients experience preventable harm due to adverse safety events, including medication errors, hospital-acquired infections, and complications from medical procedures. Notably, within the same hospitals, Black patients experienced significantly higher rates of these safety events compared to White patients of the same age and gender [14]. This disparity highlights the need to shift the focus from merely enhancing patient trust to improving the trustworthiness of healthcare organizations [13, 15]. This strategy involves not only making health systems more capable of fulfilling expectations and agreements, thereby addressing misplaced medical mistrust—skepticism towards institutions that are in fact trustworthy—but also ensures that these organizations are perceived as trustworthy [16]. Furthermore, healthcare organizations must embody trustworthiness– the ability to meet expectations and fulfill agreements– and be perceived as such. Trust in this context implies a willingness to accept risk, uncertainty and rely on the healthcare system, assuming that it will act in the patient’s best interest. Conversely, distrust signifies skepticism toward healthcare entities or providers, often rooted in past negative experiences. Thus, reducing medical mistrust and addressing distrust requires healthcare organizations to demonstrate and be acknowledged for their trustworthiness, aligning with both general positive expectations and specific patient experiences [17]. 

While trust is typically conceptualized as a patient’s willingness to rely on the healthcare system, trustworthiness is a characteristic of the system itself [18]. Conceptually, trustworthiness could be assessed through institutional metrics, such as patient safety indicators, medical error rates, policy enforcement, and transparency in decision-making. However, healthcare trustworthiness operates at multiple levels: institutions (e.g., medicine, public health, biomedical research), organizations (e.g., hospitals, clinics, insurers), and providers (e.g., doctors, nurses, pharmacists). Each of these levels may be evaluated differently by patients, as experiences with individual providers may not always align with perceptions of healthcare organizations or broader institutional structures. The distinction is important: trust reflects an individual’s readiness to be vulnerable, whereas perceived trustworthiness reflects how individuals judge the fairness, reliability, and accountability of healthcare organizations and systems [17]. Unlike patient trust, which is primarily relational and shaped by direct interactions with healthcare providers, perceived trustworthiness reflects a combination of personal experiences and broader systemic factors, including institutional behavior, public narratives, and historical context [19]. 

Given that medical mistrust significantly affects healthcare utilization, treatment adherence, self-reported health status, patient satisfaction, and participation in clinical trials—with particularly pronounced effects among racial and ethnic minorities, LGBTQ + individuals, and individuals with lower socioeconomic status—it is important to examine how trustworthiness is perceived across these groups. Differences in healthcare experiences, including exposure to bias, discrimination, and structural barriers, may shape perceived trustworthiness in distinct ways depending on whether trust is evaluated at the provider, organization, or institutional level.

This pilot study aimed to explore the feasibility and effectiveness of the Healthcare Organization and System Trustworthiness (HOST) scale as a measure of perceived trustworthiness. Prior trust measures have primarily assessed patient trust in providers or general distrust in healthcare systems, the HOST scale was designed to assess perceived institutional trustworthiness—a distinct construct that captures how individuals judge the fairness, reliability, and accountability of healthcare organizations [20–24]. Unlike interpersonal trust measures that focus on individual clinicians, and distrust measures that assess skepticism toward healthcare institutions, the HOST scale frames trustworthiness as a property of the healthcare system that is evaluated through personal experiences and broader societal perceptions [9]. Importantly, HOST integrates a forward-looking dimension, asking respondents to assess their expectations for future care encounters, rather than relying solely on retrospective assessments of past experiences.

Our goals were to (a) evaluate the factor structure of the HOST scale using exploratory and confirmatory factor analysis, (b) to evaluate the measurement invariance of the HOST across various identity and influencing factors that might affect respondents’ health behavior, and (c) assess the concurrent validity of the HOST with the frequently used and adapted Medical Mistrust Index (MMI) [25]. To evaluate concurrent validity, we examined the relationship between the HOST and MMI concerning COVID- 19 vaccine hesitancy, vaccine uptake, trust in the various actors involved in vaccine development and distribution, and trust in federal, state, and local governments. We hypothesized that the MMI and HOST scales would positively correlate because they measure related yet distinct concepts, such as discrimination versus fairness.

## Methods

We used data from the COVID- 19 Pandemic Trust, Racism, and State Violence Study, which consists of a convenience sample of Americans aged 18 years and older [26]. These data were collected through an online survey panel provided by Qualtrics Research Services between July 1 and July 26, 2021. Qualtrics collaborated with market research firms to randomly select participants who met pre-defined criteria. The original study used quota sampling to ensure representation of historically marginalized populations in the United States, with the following distribution: non-Hispanic Black (25%), Latino (25%), Asian (10%), American Indian and Alaska Native (4%), and all other groups. To ensure data quality, the original data collection process included removing duplicate entries, responses from “speeders” (participants with completion times significantly below average), nonsensical answers, and data from individuals located outside the United States. The response rate was 46.6% of 8,798 potential respondents. Respondents received compensation of $6.25, proportional to the estimated 30-minute survey completion time. The Tulane University Social Behavioral Institutional Review Board (IRB) approved our study protocol and survey instrument in accordance with the Declaration of Helsinki. Each participant completed an IRB approved consent form before participating in the study. Our final sample comprised 4,100 participants who provided complete responses for our primary outcome and demographic variables.

### Measures

#### Medical mistrust index (MMI)

**Fig. 1:**
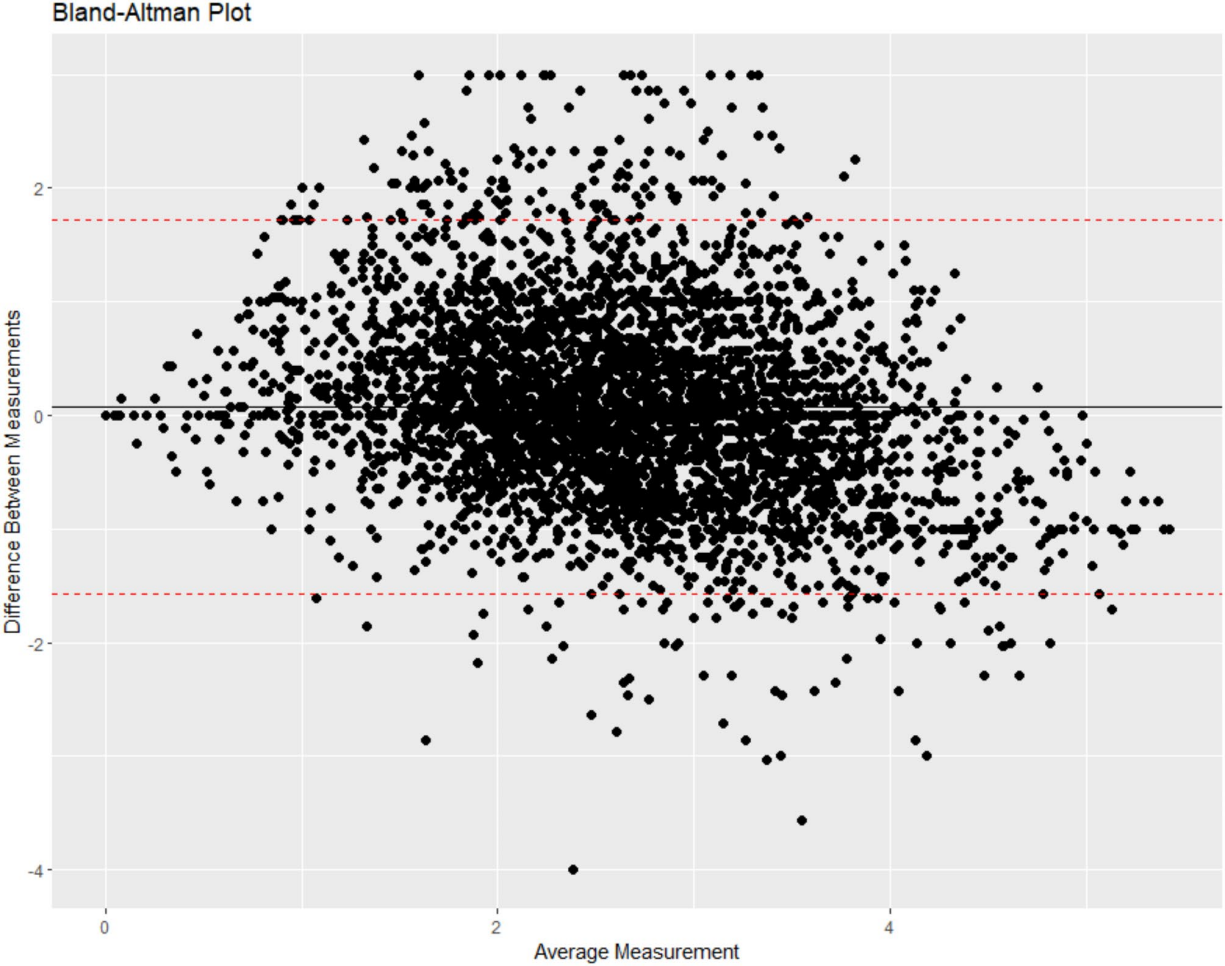
Bland-Altman plot. Note. The upper and lower boundaries of agreement (i.e., the upper and lower dashed lines) were determined by adding and subtracting 1.96 times the standard deviation of the scale score differences from the differences between the scales. These bounds represent the expected range where 95% of the data points are likely to fall. If there is considerable agreement between scale scores, the majority of differences between scale scores should fall within the 95% range

We used the short-form Medical Mistrust Index [27] which comprises seven items on a 4-point Likert scale. Participants were asked how much they agreed (i.e., 1- Strongly Disagree, 2- Disagree, 3- Agree, and 4-Strongly Agree) to a statement corresponding to suspicion of health care organizations (Fig. 1). Items included the following statements: “You’d better be cautious when dealing with healthcare organizations”; “Patients have sometimes been deceived or misled by healthcare organizations.”; “When healthcare organizations make mistakes, they usually cover it up.”; “Healthcare organizations are more concerned about making money than caring for people.” “Healthcare organizations don’t always keep your information totally private.”; “Sometimes I wonder if healthcare organizations really know what they are doing.”; and “Mistakes are common in healthcare organizations.” Each item response is assigned a score from 1 to 4, and scores are averaged across respondents. Higher scores indicate greater mistrust, and lower scores indicate lower mistrust.

#### Healthcare Organization and System Trustworthiness scale

We constructed the HOST scale by reviewing the existing literature to identify items that align with the concept of perceived trustworthiness, characterized by the perception of probabilities or expectations regarding the outcomes—whether they involve gains or losses—associated with engaging in situations that require trust, encompassing aspects of risk, uncertainty, and interdependence [19]. The HOST scale assesses individuals’ perceptions focusing on fairness, reliability, and accountability. It is not intended to measure patient trust as a behavioral disposition but rather how the healthcare system is judged based on past experiences, societal influences, and structural factors.

These studies highlight key dimensions of trustworthiness, including perceptions of fairness, reliability, and systemic discrimination, which informed the conceptual framework of the scale [8, 21, 27]. Importantly, we chose some items incorporating terms such as “me” and “my” to focus a respondent’s healthcare assessment on their own individual perspectives rather than what they thought other people might experience. Participants were asked, “How much do you agree or disagree with the following statements about healthcare institutions?” They were asked to rate their level of agreement on a 5-point Likert scale (1-Completely, 2-Mostly, 3-Somewhat, 4-Not much, and 5-Not at all) to statements that correspond to concepts of competence, fairness, and expectations of future care. Items included the following statements: “Healthcare institutions provide the highest quality medical care” (Item 1); “When treating my medical problems, healthcare institutions put my medical needs above all other considerations, including costs” (Item 2); “Healthcare institutions will be held accountable if they cause me harm” (Item 3); “Healthcare institutions treat all patients the same regardless of their race or ethnicity” (Item 4); “Health care institutions only care about keeping medical costs down, and not what is needed for my health” (Item 5). Three of the five items (1, 2, and 5) were adapted from the validated 17-item Multidimensional Trust in Health Care Systems Scale [21]. Item 4 was constructed based on findings from a systematic review of trust measures, which identified fairness as a key and often missing concept of trustworthiness in existing scales [20]. Item 3 was developed based on the concept of trustworthiness as encapsulated interest (i.e., patients trust in a person whom they believe to have strong reasons to act in their best interests) [18]. Higher HOST scale scores indicate lower ratings of trustworthiness, and lower scores indicate lower higher ratings of trustworthiness. The list of items for both the HOST scale and MMI are included in an online supplement.

### Statistical analysis

#### Exploratory and confirmatory factor analysis

To explore the factor structure of the HOST scale, we used both exploratory factor analysis (EFA) and confirmatory factor analysis (CFA) with cross-validation on a training and testing sample [28–31]. Specifically, we first performed the EFA using maximum likelihood estimation (MLE) on the training sample (*n* = 2050). Given that the response options to the HOST scale include 5-response categories, we decided to treat these as continuous, z-scored measures in the EFA and CFA. Research indicates that treating Likert items with five or more options as continuous with MLE is often acceptable in practice [32]. We followed up the EFA with CFA using MLE on the testing sample (*n* = 2050). Note that participants were randomly selected for the training and testing samples. We used the *psych* package in R to perform an EFA of the HOST [33–35]. We performed EFAs with up to 5 factors (i.e., the number of items), using both no rotation and varimax rotation with MLE. Even though a unidimensional structure was hypothesized for the HOST measures, we explored both varimax and oblimin rotation to explore the potential of multiple uncorrelated and correlated factors, respectively. We used the *lavaan* package in R to perform the CFA using MLE [36]. 

#### Measurement invariance testing

Measurement invariance refers to the extent to which the measurement properties of a latent construct remain consistent across different groups or conditions. In other words, it assesses whether the relationships between the observed indicators and the latent construct are comparable across different groups or measurement occasions. We used the forward approach to test increasing strict levels of measurement invariance (i.e., configural, metric, and scalar) for the measurement invariance testing [37, 38]. Configural, metric, and scalar invariance refer to the extent to which the measurement properties of a latent construct remain consistent across different groups of conditions. Configural invariance assesses whether the pattern of relationships between the observed indicators and latent constructs is similar across groups. Metric invariance assesses whether the factor loadings (i.e., the relationship between the observed indicators and latent construct) are equivalent across groups. Lastly, scalar invariance examines whether the factor loadings and the item intercepts (i.e., the expected item mean for someone at the mean on the latent factor) are equivalent across groups. If the measurement properties of the latent construct differ across groups, this can lead to biased or misleading conclusions. Evidence of scalar invariance is desirable because latent (or observed) scores for the HOST may be compared across groups. We tested the measurement invariance of the HOST for groupings based on gender (male, female), race (Asian, White, Black, and Hispanic), LGBTQ status (cis-gendered bisexual, gay/lesbian, and straight), and whether respondents had a regular source of care (yes, no) [29–31]. Regular source of care was defined as having a usual place, such as a doctor’s office, clinic, or health center, where one seeks medical care when sick or in need of health advice. We dropped individuals whose self-reported race was American Indian/Native American/Alaska Native and Native Hawaiian or Pacific Islander from the race measurement invariance analysis due to small sample sizes. Moreover, we dropped individuals who were missing on LGBTQ status (*n* = 48) and those who had conflicting responses as to their sexual activity and sexual orientation (*n* = 21).

In the forward approach to testing measurement invariance, the initial step involves evaluating the configural model for its suitability by employing traditional CFA fit indices [39]. While there is not a universally accepted set of criteria, indicators of satisfactory model fit typically encompass the Root Mean Square Error of Approximation (RMSEA) of ≤ 0.05, Comparative Fit Index (CFI), and Tucker-Lewis Index (TLI) ≥ 0.95 and Standardized Root Mean Square Residual (SRMR) ≤ 0.06 [40]. The following steps are to test for metric invariance and scalar invariance. Chi-squared difference tests are standard for comparing nested configural, metric, and scalar models. However, the chi-square difference tests are known to be sensitive to sample size (i.e., as sample size increases the chances of obtaining a statistically significant result). Therefore, it has been suggested that the change in goodness-of-fit indexes (ΔGOFs) is more appropriate. For example, the comparative fit index (ΔCFI) is often included with results aimed at testing for invariance across groups. In this study, we also use the ΔCFI to evaluate the comparative fit of the nested metric and scalar models where ΔCFI values of less than or equal to 0.01 indicate evidence of measurement invariance. Furthermore, we consider the ΔRMSEA where values of less than or equal to 0.015 are indicate evidence of measurement invariance. Likewise, ΔSRMR less than or equal to 0.03 (for metric invariance) and ≤ 0.015 (for scalar invariance) are indicate evidence of measurement invariance. All measurement invariance analyses were performed in a CFA framework using the *Lavaan* R package [36, 41]. 

#### Bland-Altman plot

We utilized a Bland-Altman plot, also known as a Tukey mean-difference plot to assess agreement between the HOST and the MMI [42, 43]. The Bland-Altman plot is a graphical representation that helps to evaluate the level of agreement between scale score estimates from the MMI and HOST across the entire spectrum of measurements. The Bland-Altman plot was computed as the mean difference between the MMI and HOST scale scores across the entire participant dataset. The upper and lower boundaries of the agreement were determined by adding and subtracting 1.96 times the standard deviation of the scale score differences between the scales. These bounds represent the expected range where 95% of the data points will likely fall. Given that the scales measure distinct but related constructs, we expected that there would be considerable agreement between scale scores, with the most differences between scale scores falling within the 95% range. “We use the Bland-Altman plot in addition to the concurrent validity correlation coefficients because the correlation coefficients only capture the strength of the relationship. However, they do not provide information about potential systematic biases (e.g., one measure consistently over- or underestimates values relative to the other) or individual-level differences in measurement. Therefore, we use the Bland-Altman plot to complement correlation analysis, which provides a detailed assessment of agreement between the two measures. By incorporating both correlation and Bland-Altman analyses, we provide a more comprehensive evaluation of the extent to which the two measures can be used interchangeably or whether systematic differences suggest caution in their combined use.”

#### Concurrent validity

Concurrent validity refers to the amount of agreement between two different assessments where typically one of the two assessments is new, whereas the other is known to be valid [44]. Agreement between the MMI and HOST scale on the various outcomes (i.e., here, equivalent correlations between the two scales and the criterion measures) would provide evidence of concurrent validity. To this end, we first evaluated the correlation between the two measures. Next, we assessed whether the correlations of the two measures were the same for the various criterion measures (see next section). Specifically, we evaluated two models: (1) an unconstrained model and (2) a constrained model. In the unconstrained model, the MMI and the HOST scale were allowed to predict the various criterion measures freely. In the constrained model, the MMI and the pilot measured were assumed to have the exact correlation with the criterion measures. Model comparisons were made between the constrained and unconstrained models for each criterion variable using an analysis of variance (ANOVA) framework. In this analysis, the constrained model is preferred if a difference between the unconstrained and constrained models cannot be rejected [45–47]. All analyses were performed in a CFA framework using the *lavaan* R package [36]. 

#### Criterion measures

We further used seven criterion measures to evaluate the HOST and MMI relatedness. The criterion measures related to vaccine hesitancy, vaccine receipt, and perceptions of the trustworthiness of actors involved in developing and distributing the coronavirus vaccines. The first measure corresponded to participant responses to the question, “Have you already received the COVID- 19 Vaccine?” (Yes or no). The second measure included responses to the question, “Once you are eligible to receive the vaccine in your state, how likely will you be to get it?” (1 = Strongly Disagree, 2- Disagree, 3-Agree, and 4- Strongly Agree). The third measure included responses to the question: “The benefits of the COVID- 19 vaccine outweigh the potential risks” (1 = Strongly Disagree, 2- Disagree, 3-Agree, and 4- Strongly Agree). The fourth measure included responses to the question: “How trustworthy are each of these actors which at various points have been involved in the development and distribution of the coronavirus vaccine?” This question was answered using a five-point Likert scale (1 = Completely, 2 = Mostly, 3 = Somewhat, 4 = Not much, 5 = Not at all) evaluating the following actors: (1) “Drug companies working to create and test the vaccine,” (2) “The U.S. Food and Drug Administration (FDA),” (3) “Your usual doctor or healthcare team (Skip if you don’t have one),” (4) “Pharmacies and walk-in clinics where people can get vaccinated,” and (5) “Elected officials in your state”. We modeled this measure as a latent variable where higher values indicated lower trustworthiness ratings. Lastly, we included three measures asking participants about their trust in the federal, state, and local government: (1) “What percent of the time do you think you can trust the federal government in Washington to do what is best for the country?, (2) What percent of the time do you think you can trust you state government to make decisions in a fair way, and 3)What percent of the time do you think you can trust your local government to make decisions in a fair way?”

## Results

### Descriptive statistics

Table 1 provides descriptive statistics on the analytic sample. The sample had a higher percentage of respondents who were female (51%), White (32%), cisgender straight (82%), and had a regular source of care (77%). The sample included considerable diversity in terms of race and LGBT status. 25% identified as Black/African American, 24% as Hispanic/Latinx, 13% identified as cisgender bisexual, and 5% as gay/lesbian.

**Table 1:** Sample demographic characteristics (*n* = 4,100)

Variable	Count (Percent)
Female	2087 (51.0)
Race	
American Indian/Native American/Alaska Native	163 (3.98)
Asian	407 (9.93))
Black/African American	1019 (24.85)
Hispanic/Latinx	994 (24.24)
Biracial/Multiracial	187 (4.56)
Native Hawaiian or Pacific Islander	31 (0.76)
White	1299 (31.68)
LGBTQ	
Cisgendered bisexual	540 (13.40)
Cisgendered gay/lesbian	186 (4.61)
Cisgendered straight	3305 (81.99)
No Regular Source of Care	957 (23.34)

*LGBTQ *Lesbian, Gay, Bisexual, Transgender, or Queer Identifying. Convenience sample of a national survey

### Exploratory factor analysis

The results of the EFA with varimax rotation for the training sample are reported in Table 2. The results of the EFA with oblimin are included in the online appendix. The communalities for all items of the HOST ranged from 0.4 to 0.55 except for item 5 which did not load onto any factor regardless of rotation. The communalities range between 0 and 1 and refer to the amount of variance in the item explained by the factor. In both cases, the results of the EFA provide evidence of a unidimensional factor structure based on items 1–4. The unidimensional factor accounts for over 90% of the total variance in the items regardless of rotation. In subsequent CFA analyses, we did not include item 5 due to its lack of correlation with the other HOST items. For the CFA analyses, based on the results of the EFA, we assume a unidimensional factor structure holds for items 1–4 of the HOST.

**Table 2:** Exploratory factor analysis with varimax rotation on the training sample (*n* = 2050)

	*Factor Loadings*					
	1	2	3	4	5	Communality
Item 1	0.69					0.51
Item 2	0.72					0.55
Item 3	0.60					0.40
Item 4	0.65					0.44
Item 5						0.02
Sum-of-squared loadings	1.78	0.15	0.00	0.00	0.00	
Proportion variance explained	0.92	0.08	0.00	0.00	0.00	
Cumulative variance explained	0.92	1.00	1.00	1.00	1.00	

We used maximum likelihood estimation in the psych package in R. Standardized loadings (pattern matrix) based upon correlation matrix. Factor loadings less than 0.30 not reported. The communality is the sum of the squared component loadings for the five factors extracted and represents the variance of observed variables accounted for by the factor

### Confirmatory factor analysis

The goodness-of-fit indices for the unidimensional factor model with the testing sample are reported in Table 3. All the goodness-of-fit indices indicate that the unidimensional model fits the data well. Standardized factor loadings for items 1–4 of the HOST range between 0.625 and 0.838. The standardized factor loadings indicate that all the items load highly onto the unidimensional factor. That is, the standardized factor loadings (which should not exceed 1.0) indicate better, more discriminating items (i.e., the items are good at discriminating people who have low versus high scores of the unidimensional factor). The chi-square test of model fit was not statistically significant, indicating an overall good fit (i.e., minor discrepancies between the sample and fitted covariance matrix). The CFI, TLI, RMSEA, and SRMR all had levels indicating tenable fit for the unidimensional factor.

**Table 3:** Unstandardized and standardized loadings for one-factor confirmatory factor analysis model on the test sample (*N* = 2050)

	Unstandardized (SE)	Standardized (SE)
Item 1	1.000 (0.000)	0.811 (0.010)
Item 2	1.033 (0.018)	0.838 (0.019)
Item 3	0.770 (0.020)	0.625 (0.015)
Item 4	0.869 (0.017)	0.705 (0.013)

*SE *Standard error. For the unstandardized results, item 1 is fixed to unity for identification purposes. χ^2^(2) = 1.761, *p* = 0.415; RMSEA = 0.00 [0.00, 0.42]; CFI/TLI = 1.00/1.00. SRMR = 0.006

### Measurement invariance results

The results of the measurement invariance tests for gender, race, LGBTQ status, and regular source of care are in Table 4. The results indicate that scalar invariance was achieved for gender, LGBTQ status, and a regular source of care because the change in CFI was always less than − 0.01. However, only metric invariance was achieved for race because the change in CFI from the metric to scalar invariance model was approximately − 0.02. The same conclusions were justifiable when considering the results of the change in RMSEA and SRMR. The results indicate that latent (or observed) score comparisons for the HOST are comparable across gender, LGBTQ status, and regular source of care and may be comparable across race.

**Table 4:** Measurement invariance results across various dimensions for HOST scale on test sample

**Gender**	χ^2^	df	Δχ^2^	Δdf	*p*	CFI/TLI	RMSEA	SRMR	ΔCFI
Configural	18.29	4	-	-	-	0.997/0.991	0.042	0.009	-
Metric	22.03	7	3.75	3	0.29	0.997/0.995	0.032	0.014	0.000
Scalar	90.98	10	68.95	3	< 0.01	0.984/0.981	0.063	0.030	− 0.013
**Race**	χ^2^	df	Δχ^2^	Δdf	P	CFI/TLI	RMSEA	SRMR	ΔCFI
Configural	15.76	8	-	-	-	0.998/0.995	0.032	0.008	-
Metric	29.43	17	13.67	9	0.13	0.997/0.996	0.028	0.021	− 0.001
Scalar	115.74	26	86.31	9	< 0.01	0.981/0.983	0.061	0.035	− 0.016
**LGBTQ**	χ^2^	df	Δχ^2^	Δdf	p	CFI/TLI	RMSEA	SRMR	ΔCFI
Configural	16.21	6	-	-	-	0.998/0.994	0.036	0.007	-
Metric	27.97	12	11.76	6	0.07	0.997/0.995	0.031	0.012	− 0.001
Scalar	61.39	18	33.42	6	< 0.01	0.991/0.991	0.042	0.017	− 0.006
**Regulare Care**	χ^2^	df	Δχ^2^	Δdf	p	CFI/TLI	RMSEA	SRMR	ΔCFI
Configural	10.94	4	-	-	-	0.999/0.996	0.029	0.007	-
Metric	13.39	7	2.45	3	0.48	0.999/0.998	0.021	0.009	0.000
Scalar	21.61	10	8.22	3	0.04	0.998/0.997	0.024	0.012	− 0.001

*df *Degrees of freedom, *CFI *Comparative Fit Index, *TLI *Tucker-Lewis Index, *RMSEA *Root Mean Square Error of Approximation, *SRMR *Standardized Root Mean Square Residual, *Reg*. Regular, *LGBTQ *Lesibian, Gay, Bisexual, Transgender, or Queer Identifying

### Bland-Altman plot

We examined the degree of agreement between the HOST and MMI using a Bland-Altman plot shown in Fig. 1. The plot visually indicates agreement between the scale scores resulting from the HOST and the MMI. Scale score differences on the y-axis were plotted against the average scale score values on the x-axis. The Bland-Altman plot demonstrates that the average discrepancy in scale scores between the two scales (Fig. 1 solid black line) is close to zero. This suggests that, when collapsed across the two measures, there does not appear to be substantially meaningful differences in the estimates of the scale scores.

### Concurrent validity

The primary results of the concurrent validity tests are reported and included in an online supplement. Overall, the HOST scale had evidence of concurrent validity with the MMI. The pilot measure and MMI were statistically significantly and strongly positively correlated (*r* = 0.52). The correlations between the two scales and three of the seven criterion measures were nearly identical (i.e., on the measures related to vaccine hesitancy and uptake). The lack of statistical significance between the constrained and unconstrained models evidenced the latter finding. The equivalent correlations for the measures of the criterion outcomes were: (1) vaccine receipt (*r* = 0.26), (2) likelihood of receiving the vaccine (*r* = 0.27), and (3) benefits outweigh the risks (*r* = − 0.23). However, the measures did not have equivalent correlations for four of the seven criterion variables (i.e., ratings of the trustworthiness of actors, federal, state, and local governments). The latter finding was evidenced by the statistically significant difference between the constrained and unconstrained models. For ratings of the trustworthiness of actors, the HOST was more strongly correlated (*r* = 0.71) than the MMI (*r* = 0.14). Similar findings were found in ratings of the trustworthiness of the federal (HOST *r* = − 0.38, MMI *r* = − 0.15), state (HOST *r* = − 0.40, MMI *r* = − 0.09), and local (HOST *r* = − 0.42, MMI *r* = − 0.11) governments. Note, that all of the correlation were statistically significant.

## Discussion

The primary aim of this paper was to assess the psychometric properties of the pilot HOST scale, which measures dimensions of perceived trustworthiness, such as fairness and expectations of future treatment when receiving health care. The HOST scale differs from existing trust measures in three key ways. First, while prior scales assess patient trust as a personal disposition—reflecting an individual’s willingness to rely on healthcare institutions—HOST captures perceived trustworthiness as a judgment of an institution’s fairness, reliability, and accountability. Second, whereas distrust measures like the MMI emphasize suspicion and systemic failure, HOST frames trustworthiness as a system-level characteristic that can be strengthened over time. Third, unlike static assessments of past experiences, HOST incorporates future expectations, making it particularly useful for evaluating interventions aimed at improving institutional credibility. However, HOST remains a perception-based measure—it assesses whether patients believe institutions are trustworthy rather than directly measuring observed trustworthiness (e.g., safety metrics, policy compliance).

We explored the construct of perceived trustworthiness as separate but related to medical mistrust. The beliefs that comprise medical mistrust (e.g., suspicion, discrimination, and lack of support) inform the perceived trustworthiness of healthcare organizations and systems [8]. However, medical mistrust measures like the MMI encapsulate both respondents’ personal experiences with healthcare and their beliefs about how others are treated. In contrast, they do not directly capture expectations regarding future treatment in healthcare encounters. The MMI and the HOST scale contain items corresponding to beliefs about what people think they know about the treatment of racial and ethnic minorities in the US and the competence of healthcare organizations. The HOST, however, includes items that attempt to prompt respondents to evaluate their experience when seeking care in the future (e.g., healthcare organizations will be held accountable if they cause me harm).

The HOST anchors the respondents’ evaluation of healthcare organizations and their individual experience by using the terms “my” and “me” in the item stems. For example, one item of the MMI that may be considered specific to the respondents’ experiences, “you’d better be cautious when dealing with healthcare organizations,” includes an implicit expectation that a person may experience harm when engaging with healthcare organizations. Still, it does not require an evaluation related to the respondents’ personal experiences. Instead, it prompts an assessment of what respondents think they know about people who may share one of many identities (e.g., race) with them. The item also motivates responses based on what the respondent thinks they know about an average person generally.

Item 3 of the HOST scale was piloted to capture respondents’ expectations of the care they will receive in the future. For example, the Group-Based Medical Mistrust Scale– another commonly used measure of medical mistrust– includes items that prompt respondents to describe their level of agreement with statements that assess their feelings about how the health care system treats people who are members of their ethnic group. It includes one item related to the respondents’ negative experiences with healthcare professionals but does not anchor these experiences to the expectation of future experiences. A patient may have experienced discrimination based on their race or ethnicity in the past, but it does not mean they expect the same experiences in the future. Similarly, like the MMI, the Health Care System Distrust Scale does not prompt respondents to evaluate healthcare organizations based on the expectations of the care they expect to receive [48]. The items prompt respondents to rate their level of agreement with statements about what they think they know about the healthcare system and how patients are treated generally. Unlike these other scales, the HOST scale prompts respondents to reflect on future care expectations, making it a unique measure in this regard.

The EFA revealed acceptable communalities for the HOST scale with all values ranging from 0.4 to 0.55 for items 1–4. Prior research indicates that communalities between 0.25 and 0.4 are acceptable and those above 0.7 are ideal [49]. Thus, there is evidence that the HOST scale measures a single factor. We hypothesize this single factor is a dimension of perceived trustworthiness. The results indicate that item 5 did not function as was hypothesized. We believe that the item may suffer from being double-barreled and future research should consider splitting these items into two separate statements; therefore, it was excluded in the subsequent analyses. The CFA supported a unidimensional structure relative to the HOST, which suggests that it measures the underlying construct reasonably well.

We tested the extent to which the HOST item assesses the underlying construct similarly across demographic groups. The results support measurement invariance by gender, LGBT status, and whether the respondent had a regular source of care. We tested invariance based on having a usual source of care as a proxy for experience with health care services. This is the first study to assess the measurement invariance of a trust-related measure for the LGBT population. We did not find scalar invariance by race and ethnicity by the change in CFI metric, albeit goodness-of-fit results were promising. Some racial and ethnic groups may place greater emphasis on group-based assessments of institutional trustworthiness rather than solely relying on individual experiences. Because this scale prompts respondents to reflect on their own experiences and expectations, differences in response patterns may arise based on how trustworthiness is conceptualized within different communities [8, 50]. 

Beyond this measure’s pilot phase, future research should explore this finding. Methodological research indicates that in the context of many groups, like our study’s race variable, a less restrictive change in CFI may be appropriate (i.e., − 0.02 or less). In the latter scenario, evidence of scalar invariance for race would be tenable [51]. The HOST had high concurrent validity with the MMI, which supported our initial hypothesis. However, the HOST was more strongly correlated with our criterion measures of the trustworthiness of actors involved in developing and distributing the COVID- 19 vaccines. The finding suggests that although the HOST captures similar concepts as the MMI– and is as predictive of vaccine hesitancy and uptake– it assesses a construct separate from the MMI, which we propose is a dimension of perceived trustworthiness. The HOST is also potentially more usable than the MMI in real-world settings because it achieves similar predictive ability with fewer items.

Overall, the finding supports our hypothesis that medical mistrust and perceived trustworthiness are related but distinct concepts. Our findings also suggest that framing items in the context of personal expectations of the future may play a role in predicting the perceived trustworthiness of people and institutions involved in the development and distribution of the coronavirus vaccines and overall trust in federal, state, and local government. Researchers should consider the framing of their items when using or adapting existing trust measures in other contexts.

### Limitations

Our analysis had some limitations. First, we used a convenience sample from market research panels. We could not assess the respondents’ health literacy, which could influence their ability to interpret our items. There may have also been a higher likelihood of participation from people interested in health-related issues. The results may not represent the true distribution of attitudes within populations outside our sample. Our study focused on assessing medical mistrust and perceived trustworthiness in the context of developing and distributing COVID- 19 vaccines. These findings may not hold to the same degree in another context. The pilot HOST scale was not developed using cognitive testing or other best practices for developing scales which could have led to response bias [52]. However, all except two items of the HOST (i.e., healthcare institutions will be held accountable if they cause me harm) were adapted from the Multidimensional Trust in Health Care Systems Scale, which was developed on a review of the literature, focus groups, and expert opinion [21]. The HOST scale, while not encompassing all possible dimensions of trustworthiness such as the quality and nature of communication, effectively aligns with crucial aspects like competence, reliability, and honesty. Still, the items do align with other key dimensions of trustworthiness such competence, reliability, and honesty [20]. Additionally, we contend that its unique emphasis on future expectations serves as the primary distinguishing feature from other trust-related constructs. This focus on anticipatory trust differentiates the HOST, highlighting its innovative approach to measuring trustworthiness.

## Conclusion

Our paper presents a novel and concise instrument, the HOST scale, that captures perceived trustworthiness of healthcare organizations, distinct from medical mistrust and patient trust. Our evidence suggests the host scale has adequate factor structure, reliability, validity, and measurement invariance across different groups. The MMI and the HOST scale performed similarly when predicting vaccine hesitancy and receipt; however, the HOST scale was more predictive of trustworthiness ratings of healthcare institutions, policymakers, and government agencies.

Our findings have both theoretical and practical implications. While increasing patient trust is often seen as a way to improve engagement, our results highlight the need for healthcare institutions to demonstrate and maintain trustworthiness. Perceived trustworthiness of healthcare organizations shapes healthcare engagement beyond individual provider relationships. Future research should examine its interaction with provider trust. Measures incorporating the HOST scale’s approach—assessing expectations for future care experiences—could help evaluate health communication efforts and interventions aimed at enhancing trustworthiness. The findings from this pilot study can also inform refinements to trustworthiness measurement through expert input, cognitive testing, and additional psychometric evaluation.

## Supplementary Information

Supplementary Material 1.

## Data Availability

The datasets analyzed during the current study are available upon request from the corresponding author.
