# Supplementary material for: Pilot evaluation of the Health Organization and System Trustworthiness scale: reliability and validity testing
Source: BMC Health Serv Res. 2025 May 21;25:739. doi: 10.1186/s12913-025-12724-7 (PMC12093774; doi:10.1186/s12913-025-12724-7)
Supplement: Supplementary file 1 — Supplementary Material 1. [file 12913_2025_12724_MOESM1_ESM.docx]

**Supplement Table 1.** Development and Sources of the Healthcare Organization and System Trustworthiness (HOST) Scale Items

| **HOST Scale Item** | **Adapted or New** | **Original Statement (if adapted)** | **Relevant Literature** | **Justification** |
| --- | --- | --- | --- | --- |
| **Item 1: Healthcare institutions provide the highest quality medical care.** | Adapted from the Multidimensional Trust in Health Care Systems Scale | "Healthcare institutions provide the highest quality in medical care." | Egede & Ellis (2008), Armstrong et al. (2008), LaVeist et al. (2009) | Quality of care is a foundational component of trustworthiness, influencing perceptions of system reliability and competence. |
| **Item 2: When treating my medical problems, healthcare institutions put my medical needs above all other considerations, including costs.** | Adapted from the Multidimensional Trust in Health Care Systems Scale | "When treating my medical problems, health care institutions put my medical needs above all other considerations, including costs." | LaVeist et al. (2009), Benkert et al. (2019) | Trustworthiness is shaped by perceptions of whether healthcare institutions prioritize patient well-being over financial incentives, reinforcing fairness and ethical responsibility. |
| **Item 3: Healthcare institutions will be held accountable if they cause me harm.** | New Item | N/A | Shea et al. (2008), Armstrong et al. (2008), Benkert et al. (2019) | Encapsulated interest suggests that individuals trust institutions when they believe the institution has a vested interest in acting in their best interest (Hardin, 2003). Accountability is a key dimension of trustworthiness, ensuring that healthcare institutions recognize, disclose, and correct harm in ways that reinforce confidence in their reliability and ethical commitments. |
| **Item 4: Healthcare institutions treat all patients the same regardless of their race or ethnicity.** | Adapted from the Revised Health Care System Distrust Scale | "Patients get the same medical treatment from the Health Care System, no matter what the patient’s race or ethnicity." | Egede & Ellis (2008), Shea et al. (2008), LaVeist et al. (2009), Armstrong et al. (2008) | Perceived fairness in healthcare interactions is a key driver of trustworthiness. Racial and ethnic disparities in healthcare outcomes have been widely documented, making race/ethnicity a critical axis for measuring fairness. |
| **Item 5: Healthcare institutions only care about keeping medical costs down, and not what is needed for my health.** | Adapted from the Multidimensional Trust in Health Care Systems Scale, the Revised Health Care System Distrust Scale, and the Medical Mistrust Index | "Health care institutions only care about keeping medical costs down, and not what is needed for my health." / "The Health Care System puts making money above patients’ needs." / "Healthcare organizations are more concerned about making money than taking care of people." | Egede & Ellis (2008), Armstrong et al. (2008), LaVeist et al. (2009) | Economic motivations can undermine trustworthiness when patients perceive that financial concerns outweigh patient care priorities. Transparency in financial decision-making reinforces public trust. |

**Supplement Table 2.** Medical Mistrust Index (MMI): Original and Study-Used Items

| **Item Number** | **Original MMI Statement (LaVeist et al., 2009)** | **Study-Used MMI Statement** |
| --- | --- | --- |
| Item 1 | "You’d better be cautious when dealing with health care organizations." | "You’d better be cautious when dealing with healthcare organizations." |
| Item 2 | "Patients have sometimes been deceived or misled by health care organizations." | "Patients have sometimes been deceived or misled by healthcare organizations." |
| Item 3 | "When health care organizations make mistakes, they usually cover it up." | "When healthcare organizations make mistakes, they usually cover it up." |
| Item 4 | "Health care organizations have sometimes done harmful experiments on patients without their knowledge." | [Item not included in study] |
| Item 5 | "Health care organizations don’t always keep your information totally private." | "Healthcare organizations don’t always keep your information totally private." |
| Item 6 | "Sometimes I wonder if health care organizations really know what they are doing." | [Item not included in study] |
| Item 7 | "Mistakes are common in health care organizations." | "Mistakes are common in healthcare organizations." |

**Supplement Table 3.** Exploratory factor analysis with oblimin rotation on the training sample (n =2050)

|  | Factor | | | | |  |
| --- | --- | --- | --- | --- | --- | --- |
|  | 1 | 2 | 3 | 4 | 5 | Communality |
| Item 1 | 0.71 |  |  |  |  | 1.00 |
| 0Item 2 | 0.75 |  |  |  |  | 1.00 |
| Item 3 | 0.62 |  |  |  |  | 1.00 |
| Item 4 | 0.67 |  |  |  |  | 1.00 |
| Item 5 |  |  |  |  |  | 1.20 |
|  |  |  |  |  |  |  |
| Sum-of-squared loadings | 1.90 | 0.02 | 0.00 | 0.00 | 0.00 |  |
| Proportion variance explained | 0.99 | 0.01 | 0.00 | 0.00 | 0.00 |  |
| Cumulative variance explained | 0.99 | 1.00 | 1.00 | 1.00 | 1.00 |  |

*Note.* We used maximum likelihood estimation in the psych package in R. Standardized loadings (pattern matrix) based upon correlation matrix. Factor loadings less than 0.30 not reported. The communality is the sum of the squared component loadings for the five factors extracted and represents the variance of observed variables accounted for by the factor.

**Supplement Table 4.** Concurrent validity testing

| **Vaccine Receipt** | df | χ^2^ | Δχ^2^ | p |
| --- | --- | --- | --- | --- |
| Unconstrained Model | 52 | 136.54 | - | - |
| Constrained Model | 53 | 141.19 | 3.18 | .07 |
| **Vaccine Likelihood** | df | χ^2^ | Δχ^2^ | p |
| Unconstrained Model | 52 | 140.36 | - | - |
| Constrained Model | 53 | 140.45 | 0.06 | .80 |
| **Vaccine Worth it** | df | χ^2^ | Δχ^2^ | P |
| Unconstrained Model | 52 | 154.80 | - | - |
| Constrained Model | 53 | 155.18 | 0.27 | .60 |
| **Trustworthiness Actors** | df | χ^2^ | Δχ^2^ | P |
| Unconstrained Model | 116 | 1610 | - | - |
| Constrained Model | 117 | 1892 | 281.97 | <.01 |
| **Trust Federal** |  |  |  |  |
| Unconstrained Model | 52 | 495.27 | - | - |
| Constrained Model | 53 | 539.15 | 43.88 | <.01 |
| **Trust State** |  |  |  |  |
| Unconstrained Model | 52 | 466.43 | - | - |
| Constrained Model | 53 | 546.23 | 79.80 | <.01 |
| **Trust Local** |  |  |  |  |
| Unconstrained Model | 52 | 467.20 | - | - |
| Constrained Model | 53 | 551.17 | 83.97 | <.01 |

*Note.* df – degrees of freedom. In the unconstrained model, the MMI and the HOST scale were allowed to freely predict the various criterion measures. In the constrained model, the MMI and the pilot measured were assumed to have the same correlation with the criterion measures.
